# The intron-enriched HERV-K(HML-10) family suppresses apoptosis, an indicator of malignant transformation

**DOI:** 10.1186/s13100-016-0081-9

**Published:** 2016-12-07

**Authors:** Felix Broecker, Roger Horton, Jochen Heinrich, Alexandra Franz, Michal-Ruth Schweiger, Hans Lehrach, Karin Moelling

**Affiliations:** 1 Max Planck Institute for molecular Genetics, Ihnestr. 63-73, 14195 Berlin, Germany; 2 Institute of Medical Microbiology, University of Zurich, Gloriastr. 32, 8006 Zurich, Switzerland; 3 Current affiliation: Max Planck Institute of Colloids and Interfaces, Am Mühlenberg 1, 14424 Potsdam, Germany; 4 Current affiliation: University of Zurich, Institute of Molecular Life Sciences, Winterthurerstr. 190, 8057 Zurich, Switzerland; 5 Current affiliation: Functional Epigenomics, CCG, Cologne University Hospital, University of Cologne, Weyertal 115b, 50931 Cologne, Germany; 6 Dahlem Centre for Genome Research and Medical Systems Biology, Fabeckstr. 60-62, 14195 Berlin, Germany

**Keywords:** Apoptosis, Cancer, DAP3, Death-associated protein 3, Endogenous retrovirus, Gene regulation, Genome evolution, HERV, HERV-K(HML-10)

## Abstract

**Background:**

Human endogenous retroviruses (HERVs) constitute 8% of the human genome and contribute substantially to the transcriptome. HERVs have been shown to generate RNAs that modulate host gene expression. However, experimental evidence for an impact of these regulatory transcripts on the cellular phenotype has been lacking.

**Results:**

We characterized the previously little described HERV-K(HML-10) endogenous retrovirus family on a genome-wide scale. HML-10 invaded the ancestral genome of Old World monkeys about 35 Million years ago and is enriched within introns of human genes when compared to other HERV families. We show that long terminal repeats (LTRs) of HML-10 exhibit variable promoter activity in human cancer cell lines. One identified HML-10 LTR-primed RNA was in opposite orientation to the pro-apoptotic Death-associated protein 3 (*DAP3*). In HeLa cells, experimental inactivation of HML-10 LTR-primed transcripts induced *DAP3* expression levels, which led to apoptosis.

**Conclusions:**

Its enrichment within introns suggests that HML-10 may have been evolutionary co-opted for gene regulation more than other HERV families. We demonstrated such a regulatory activity for an HML-10 RNA that suppressed DAP3-mediated apoptosis in HeLa cells. Since HML-10 RNA appears to be upregulated in various tumor cell lines and primary tumor samples, it may contribute to evasion of apoptosis in malignant cells. However, the overall weak expression of HML-10 transcripts described here raises the question whether our result described for HeLa represent a rare event in cancer. A possible function in other cells or tissues requires further investigation.

**Electronic supplementary material:**

The online version of this article (doi:10.1186/s13100-016-0081-9) contains supplementary material, which is available to authorized users.

## Background

About half of the human genome is composed of transposable elements (TEs) [1], and recent evidence suggests even a fraction of up to two thirds [2]. The most abundant TEs in the human genome are retroelements (REs) that amplify via a ‘copy-and-paste’ mechanism involving reverse transcription of an RNA intermediate [1, 3].

One class of REs, HERVs, comprises remnants of ancient retroviral germ line cell infections that became evolutionary fixed in the genome. About 450,000 HERV elements constitute 8% of the human genome and are classified into about 30 families [1, 4]. HERVs are structurally similar to proviruses of present-day retroviruses where the *gag*, *pol* and *env* genes are flanked by two long terminal repeats (LTRs) that act as promoters [4]. HERVs and other REs have been shown to influence gene regulation by providing regulatory elements such as enhancers, promoters, splice- and polyadenylation sites, for various host genes [3]. REs of all classes often contain functional promoters and consequently contribute to a large fraction of the human transcriptome [5]. Numerous REs are located within introns of host genes and might be involved in antisense gene regulation in *cis* [1]. The potential significance of RE-mediated *cis*-antisense gene regulation is suggested by the genome-wide presence of about 48,000 transcription start sites (TSSs) within HERVs and other REs that are in reverse orientation to overlapping host genes [6].

Promoter activity, a prerequisite for REs to exert antisense-mediated gene regulation, has been shown for representative LTRs of HERV-E [7], HERV-W [8], HERV-H [9–12], HERV-L [9], HERV-I [13] and HERV-K(HML-2), HML standing for human mouse mammary tumor virus-like [14–17]. The latter HERV family, HML-2 in the following, is the phylogenetically most recent and most active one in the human genome [3, 4], with about 50% of LTRs being transcriptionally active [15]. Antisense gene regulation in *cis* has been shown for HML-2 LTRs located within introns of the *SLC4A8* (a sodium bicarbonate co-transporter) and *IFT172* (intraflagellar transport protein 172) genes [14]. In addition, the *PLA2G4A* gene that encodes a phospholipase with a possible implication in tumorigenesis is negatively regulated by a HERV-E LTR-primed transcript [7]. These three individual cases are presently the only experimentally verified examples of the influence of LTR-primed transcripts on gene regulation.

A HERV family phylogenetically related to HML-2 is HERV-K(HML-10), HML-10 in the following [4]. The prototypical HML-10 provirus located within an intron of the long variant of the Complement Component 4 (*C4*) gene has been shown to possess promoter activity in its 3′LTR [18, 19]. Since this provirus remains the only one studied in detail to date, we here characterized the HML-10 family in more detail. We found that HML-10 invaded the ancestral genome of the Old World monkey (OWM) lineage about 35 Mya. A survey of the human genome revealed that HML-10 sequences were significantly enriched within host gene introns, indicating their evolutionary recruitment for gene regulatory functions. Three intron-located HML-10 proviruses exerted LTR promoter activity in the human HEK293T and HepG2 tumor cell lines in vitro. Transcriptional orientation and strength varied substantially between the cell lines and promoter activity was suppressed by interferon-gamma (IFNγ). One of the proviral LTRs showed transcriptional activity in opposite orientation to the encompassing pro-apoptotic *DAP3* gene that encodes a signaling protein of the Death Receptor (DR) pathway [20, 21]. We provide evidence that HML-10 LTR-primed transcripts negatively regulate *DAP3* expression in HeLa cells, as their inactivation by antisense oligonucleotides (ASOs) led to a 10-fold increase in *DAP3* mRNA levels and efficiently promoted apoptosis. Our findings support the functional relevance of LTR-primed *cis*-regulatory transcripts for human gene regulation and the cellular phenotype and function.

## Results

### HML-10 elements are 35 million years old and enriched within human genes

To identify potential priming of *cis*-acting regulatory transcripts by HERVs, we mined the GRCh38/hg38 human genome assembly [1] for sequences of the previously little described HML-10 family. The prototype member of HML-10 is an intron-located provirus in the long form of the *C4* gene that exhibits LTR promoter activity in vitro [18, 19]. Expression of this provirus has been detected via microarray before, for instance, in brain, breast, kidney and skin tissue, blood cells as well as various human cancer cell lines [22–27].

**Fig. 1:**
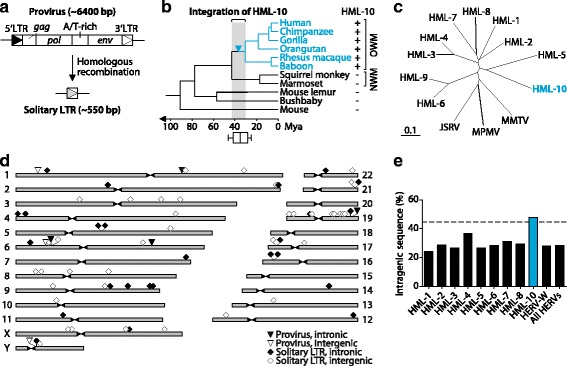
Characteristics of the HML-10 endogenous retrovirus family. **a** Structure of HML-10 proviruses [18]. **b** Estimation of the evolutionary age of HML-10 with divergence times as reported before [78]. The *box*-and-*whiskers* plot shows age estimation by sequence comparison of LTRs from six complete proviruses (elements nos. 1, 3, 20, 22, 25 and 68 in Table 1) in the human genome [28]. The *arrowhead* indicates the integration events in the OWM lineage. **c** Neighbor-joining tree of Pol protein sequences of different endogenous and exogenous betaretroviruses [18, 72]. The *horizontal bar* represents 0.1 substitutions per amino acid position. **d** Chromosomal distribution of HML-10 elements in the human genome. Details can be found in Table 1. **e** Comparison of genomic fractions of intragenic elements (located within the boundaries of RefSeq [33] genes) between HML-10 and other HERV families in the human genome. All observed distributions differed significantly from the expected value for random integration that is shown as *dotted horizontal line*, with *P*-values ≤ 0.01 inferred by chi-square tests

The provirus inside of the *C4* gene is currently the only HML-10 sequence described in the literature [18, 19]. With a size of about 6400 basepairs (bp) it contains the retroviral *gag*, *pol* and *env* genes, an A/T-rich stretch of unknown function between *pol* and *env* and two flanking LTRs [18] (Fig. 1a). Most HERV elements found in the human genome today have undergone homologous recombination between their two proviral LTRs, leaving behind solitary LTRs [1, 3, 4] that in this case have a size of about 550 bp. We identified seventy HML-10 elements within the human genome (Table 1). Of these, seven are proviruses with the structure 5′LTR-*gag*-*pol*-A/T-rich-*env*-3′LTR (with element no. 58 lacking the 5′LTR) and 63 are solitary LTRs. Some of the elements are truncated at either end or harbor other REs, mostly Alus. HERV sequences can be amplified by chromosomal duplication events following integration [4]. To reveal whether the identified HML-10 elements represent independent integration events, we determined their target site duplications (TSDs). The TSDs were expected to differ between independently acquired HML-10 elements. It has been shown previously that the provirus in the *C4* gene (element no. 22) created a 6 bp TSD [18]. Confirming these findings, we could identify TSDs of 5 or 6 bp for most (59 of 70) HML-10 elements (Table 1). All identified TSDs had a unique sequence, whereby the two copies of element no. 22 showed an identical 6 bp TSD with the expected sequence [18]. Alignment of the flanking regions of each HML-10 element (±1000 bp) revealed no sequence homology except for the two proviruses of element no. 22 as well as between elements nos. 27 and 45 (Additional file 1: Figure S1). Thus, one of the latter two has arisen through chromosomal duplication and the other 69 HML-10 elements listed in Table 1 are likely the result of independent retroviral integration events.

**Table 1:** HML-10 elements in the human genome

No.	Chr.band	Coordinates (strand)	Structure	TSD	Integrated REs	Position	RefSeq genes (rel. orientation)	RefSeq genes ± 10,000 bp
1	1p36.13	chr1:19926886-19932710 (-)	Provirus	WWAAAT	-	Intergenic	-	*PLA2G2E*
2	1p35.3	chr1:29156730-29157306 (-)	LTR	GTTAC	-	Intronic	*SFRS4* (s)	-
3	1q22	chr1:155691832-155699521 (-)	Provirus	ATTAAG	AluSp; MER11B	Intronic	*DAP3* (as)	*YY1AP1*
4	1q23.1	chr1:158534566-158535143 (+)	LTR	GTCCAA	-	Intergenic	-	-
5	1q32.3	chr1:213045116-213046160 (-)	LTR^a^	TAGTGG	-	Intergenic	-	*RPS6KC1*
6	2p11.2	chr2:86245199-86245755 (+)	LTR	TATAC	-	Intronic	*REEP1* (as)	-
7	2q37.3	chr2:238659222-238659778 (+)	LTR	not found	-	Intergenic	-	-
8	2q37.3	chr2:240556198-240556747 (+)	LTR	not found	-	Intronic	*ANKMY1* (as)	*DUSP28*
9	3p14.2	chr3:58724279-58724839 (+)	LTR	CAGCAG	-	Intergenic	-	-
10	3q12.2	chr3:101089113-101089658 (+)	LTR	AGGCAC	-	Intergenic	-	-
11	3q21.3	chr3:128828801-128835173 (+)	LTR	TGCAT	AluY; LTR7B; HERVH	Intergenic	-	-
12	3q24	chr3:146283898-146284161 (-)	LTR^b^	not found	-	Intergenic	-	-
13	4p16.3	chr4:1709255-1710065 (+)	LTR	ATGGGG	AluY	Intronic	*SLBP* (as)	*TMEM129*
14	4p16.1	chr4:8441728-8442282 (-)	LTR	YYTTTA	-	Intronic	*TRMT* (as)	*ACOX3*
15	4q31.21	chr4:143777776-143778330 (+)	LTR	TCARCC	-	Intergenic	-	-
16	5q14.1	chr5:78245022-78245575 (-)	LTR	TCYWCA	-	Intronic	*AP3B1* (s)	-
17	5q14.1	chr5:78248004-78248093 (-)	LTR^b^	not found	-	Intronic	*AP3B1* (s)	-
18	5q31.3	chr5:142088759-142089306 (-)	LTR	TTGGTG	-	Intergenic	--	-
19	6p24.1	chr6:12989575-12990131 (-)	LTR	GAAAAC	-	Intronic	*PHACTR1* (as)	-
20	6p22.1	chr6:27187520-27196279 (+)	Provirus	AAGATM	3× AluY; AluYc; LTR13A	Intergenic	-	-
21	6p22.1	chr6:28607908-28608446 (+)	LTR	CATGTT	-	Intergenic	-	-
22	6p21.33	chr6:31984691-31991052 (-) chr6:32017429-32023790 (-)	Provirus	TGTCTG TGTCTG	-	Intronic	*C4A*/*C4B* (as)	*CYP21A1P*; *STK19*; *TNXB*
23	6p21.32	chr6:32512829-32513385 (+)	LTR	GGGGRG	-	Intergenic	-	*HLA-DRB5*
24	6q21	chr6:114011436-114018365 (+)	LTR	CCCTAT	LTR7B; HERVH	Intergenic	-	-
25	6q22.31	chr6:122504844-122512093 (-)	Provirus	GGACAT	3× AluY	Intronic	*PKIB* (as)	-
26	7q36.2	chr7:154936774-154937317 (-)	LTR	ACTCCA	-	Intronic	*PAXIP1* (as)	*PAXIP1-AS2*
27	8p22	chr8:17915846-17916036 (-)	LTR^b^	CCCMTA	-	Intergenic	-	*PCM1*
28	8p21.3	chr8:22985089-22985644 (-)	LTR	CCTCYY	-	Intergenic	-	*RHOBTB2*
29	8q11.1	chr8:46533684-46534254 (+)	LTR	CATTTC	-	Intergenic	-	-
30	8q21.13	chr8:82206225-82206776 (+)	LTR	CASCCK	-	Intergenic	-	-
31	9p13.3	chr9:34539821-34540365 (-)	LTR	GGCATG	-	Intergenic	-	-
32	9q22.1	chr9:87984145-87984721 (-)	LTR	TATGGC	-	Intergenic	-	*CDK20*
33	9q22.31	chr9:92523403-92523958 (-)	LTR	WATTGT	-	Intronic	*CENPP* (as); *ECM2* (s)	-
34	9q31.3	chr9:109072071-109072632 (+)	LTR	CMAAAG	-	Intronic	*TMEM245* (as)	-
35	9q34.11	chr9:129101280-129101834 (+)	LTR	GGGGAA	-	Intronic	*CRAT* (as)	*PPP2R4*
36	9q34.13	chr9:132289026-132289571 (+)	LTR	CTCTYA	-	Intronic	*SETX* (as)	-
37	10p11.21	chr10:37685164-37685717 (+)	LTR	GAATC	-	Intergenic	-	-
38	11p11.2	chr11:43747422-43747974 (+)	LTR	GTTCTG	-	Intronic	*HSD17B12* (s)	-
39	12p13.1	chr12:12810288-12810845 (-)	LTR	ATCTA	-	Intergenic	-	*DDX47*
40	12q24.33	chr12:132949015-132949570 (+)	LTR	GTATC	-	Intronic	*ZNF605* (as)	-
41	13q13.3	chr13:37090225-37090778 (+)	LTR	CCTGTT	-	Intergenic	-	-
42	14q31.1	chr14:79706443-79707024 (+)	LTR	TTGGTC	-	Intronic	*NRXN3* (s)	-
43	16p13.13	chr16:10785748-10785943 (+)	LTR^b^	not found	-	Intronic	*FAM18A* (as)	-
44	16p13.13	chr16:10788495-10789043 (-)	LTR	GAGAYC	-	Intronic	*FAM18A* (s)	-
45	17p13.1	chr17:8056835-8057031 (+)	LTR^b^	not found	-	Intergenic	-	*ALOX15B*
46	17p13.1	chr17:8082291-8082848 (+)	LTR	CCAGG	-	Intronic	*ALOX12B* (as)	*MIR4314*
47	17q11.2	chr17:32913177-32913734 (+)	LTR	GGTATR	-	Intergenic	-	-
48	19p13.3	chr19:2863773-2863865 (+)	LTR^b^	not found	-	Intergenic	-	*ZNF555*; *ZNF556*
49	19p13.2	chr19:7100429-7100974 (-)	LTR	GTCTC	-	Intergenic	-	-
50	19p12	chr19:21987441-21988020 (+)	LTR	ATAAYA	-	Intronic	*ZNF208* (as)	-
51	19p12	chr19:23876577-23877146 (-)	LTR	CTCCCC	-	Intergenic	-	-
52	19q13.11	chr19:34595165-34595718 (-)	LTR	TGTAGG	-	Intergenic	-	*SCGBL*
53	19q13.12	chr19:36636542-36637090 (-)	LTR	not found	-	Intergenic	-	*ZNF382*; *ZNF461*
54	19q13.2	chr19:42667555-42668111 (-)	LTR	GTGTG	-	Intergenic	-	-
55	19q13.31	chr19:44283216-44283766 (-)	LTR	GTAAG	-	Intergenic	-	*ZNF233*; ZNF*235*
56	19q13.32	chr19:46030891-46031467 (+)	LTR	CAAGGT	-	Intergenic	-	*IGFL4*; *PGLYRP1*
57	19q13.41	chr19:51900477-51900716 (-)	LTR^b^	not found	-	Intronic	*ZNF649* (s); *ZNF619* (as)	*ZNF649-AS1*
58	19q13.41	chr19:52460866-52466497 (-)	Provirus^b^	AAAAC	-	Intronic	*ZNF578* (as)	*ZNF534*
59	21q22.3	chr21:43698519-43699073 (+)	LTR	TTTAG	-	Intergenic	-	*RRP1B*
60	21q22.3	chr21:43738447-43739417 (+)	LTR	CTAAT	AluSx; AluY	Intronic	*PDXK* (as)	-
61	22q11.21	chr22:20818927-20819480 (-)	LTR	TAAGA	-	Intronic	*PI4KA* (s)	-
62	22q13.31	chr22:45188740-45189285 (+)	LTR	TGCAAC	-	Intergenic	-	*NUP50*; *KIAA0930*
63	Xp11.23	chrX:48047190-48047228 (-)	LTR^b^	not found	-	Intergenic	-	*ZNF630-AS1*
64	Xp11.22	chrX:51636506-51637062 (+)	LTR	GCTCTA	-	Intergenic	-	-
65	Xq22.2	chrX:103469871-103470407 (-)	LTR	GRGGAG	-	Intergenic	--	--
66	Xq22.3	chrX:107232277-107232369 (-)	LTR^b^	not found	-	Intronic	*PIH1D3* (as)	-
67	Xq27.1	chrX:139517757-139518302 (+)	LTR	CTTAAG	-	Intergenic	-	-
68	Yq11.221	chrY:12993871-13001093 (-)	Provirus	TGSATT	AluY; LTR2B	Intergenic	-	-
69	Yq11.221	chrY:13333756-13334302 (+)	LTR	CRYAGC	-	Intronic	*UTY* (as)	-
70	Yq11.221	chrY:16492757-16493302 (+)	LTR	TCCAAR	-	Intergenic	-	-

Chromosome bands and coordinates are according to the hg38 assembly [1] and RepeatMasker annotation [70]

As, antisense orientation; s, sense orientation; TSD, target site duplication; ^a^denotes a tandemly repeated LTR; ^b^denotes truncated elements.

Ambiguous nucleotides are indicated as follows: K, G or T; M, A or C; S, C or G; R, A or G; W, A or T; Y, C or T

To reveal the evolutionary history of HML-10, we first searched for HML-10 sequences in genomes of different mammalian species. HML-10 was identified in all investigated genomes of the OWM lineage, but was absent in the genomes of New World monkeys (NWMs) and the more distantly related species mouse lemur, bushbaby and mouse (Fig. 1b). The OWM genomes contained between 80 and 96 HML-10 sequences (Additional file 2: Table S1). Of note, about 600 sequences annotated as HML-10 by RepeatMasker were found in the investigated NWM genomes that however shared little sequence homology with the ones found in OWMs. Thus, the annotated HML-10 elements in OWM and NWM genomes likely represent two distinct HERV families.

**Fig. 2:**
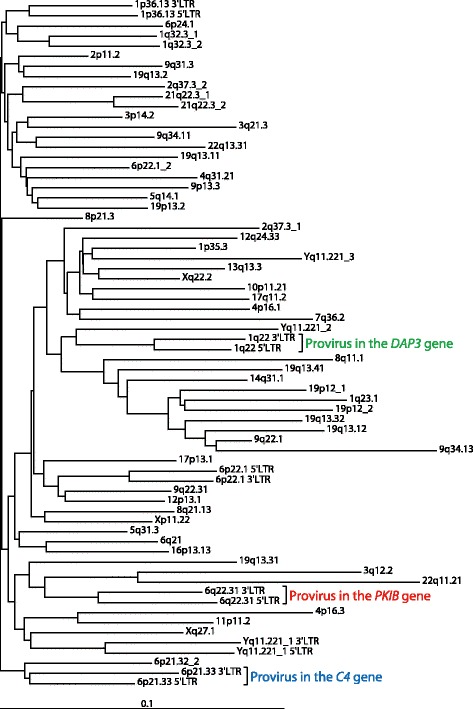
Neighbor-joining tree of 68 complete HML-10 LTRs in the human genome. HML-10 LTR sequences (see Table 1) were retrieved from the human genome GRCh38/hg38 assembly [1] according to RepeatMasker [70] annotation. The horizontal bar represents 0.1 substitutions per nucleotide position

The evolutionary age of HML-10 was estimated by calculating the nucleotide sequence divergence between both LTRs of each of the six complete proviruses (Table 1), applying a mutation rate of 2.28 substitutions per site and year × 10^−9^ as described [28]. This analysis yielded an evolutionary age of 35.3 ± 7.8 million years (mean ± SD, see box-and-whiskers plot in Fig. 1b). Phylogenetic neighbor-joining analysis of 68 complete human HML-10 LTRs, including both LTRs of each of six complete proviruses, revealed a near-monophyletic tree (Fig. 2), indicating a single integration period. Therefore, the infectious progenitor of HML-10 likely invaded the ancestral genomes of OWM during a brief period around 35 Mya (Fig. 1b). The same age has been attributed before to other endogenous human betaretrovirus families, including HML-2 [4], HML-3 [29], HML-4 [30] and HML-6 [31]. In contrast, the HML-5 infectious progenitor was active about 55 Mya [32] and HML-2 has remained active after the divergence of humans and chimpanzees about six Mya [4]. Neighbor-joining analysis of *pol* sequences of various endogenous and exogenous betaretroviruses showed that HML-10 is closely related to HML-1 through HML-9 HERVs and the extant exogenous retroviruses JSRV (Jaagsiekte sheep retrovirus), MPMV (Mason-Pfizer monkey virus) and MMTV (mouse mammary tumor virus) (Fig. 1c).

HML-10 elements are non-randomly distributed among human chromosomes (Fig. 1d). Most notably, the relatively small chromosome 19 harbored the highest number of elements (11 of 70). This was a first indication that HML-10 sequences were preferentially located near host genes, since chromosome 19 is the most gene-dense one [1]. Of the 70 HML-10 elements, 29 (41.4%) were found within introns of human genes (as annotated by RefSeq [33]), and 16 of the remaining 41 intergenic elements were located in proximity (±10,000 bp) to at least one RefSeq gene (Table 1). The relatively frequent location of HML-10 in the vicinity of host genes is a feature that is not shared with other HERV families studied in this regard. Namely, only 28% of all HERV-W elements are located within introns of genes [8] and HML-2 was found to be enriched outside genes, although *de novo* infection and integration of a resurrected HML-2 retrovirus favored actively transcribed regions [34], a common feature of present-day retroviruses as well [35]. Based on the published literature about HERV-W and HML-2, we compared the integration preferences of these two HERV families with HML-10 as well as all other HML families, except for HML-9 that was not annotated by RepeatMasker, at the genome-wide level. HML-10 sequences were found with higher frequency within the boundaries of RefSeq genes (47.94%) than expected by random distribution (44.79%), whereby sequences of HML-2 (28.76%), HERV-W (27.95%) and of all annotated HERV elements combined (28.46%) were less abundant within genes (Fig. 1e). The intragenic sequence fractions of the other HML families were below the expected value for random integration and ranged between 24.35% (HML-1) and 36.75% (HML-4). Thus, the frequent location of HML-10 within host genes appears to be a unique feature of this family and suggests an important and conserved function for gene regulation. The intronic HML-10 elements showed a distinct bias for integration in reverse orientation relative to the respective encompassing gene, with 20 being in reverse (antisense) and 7 in parallel (sense) orientation (Table 1). Two elements were reverse to one gene and parallel to another overlapping one. The integration bias of HML-10 indicates that the reverse orientation was evolutionarily favored, which is in line with previous findings of other HERV families [36–38]. One explanation for this observation is that parallel intronic proviruses are more likely to disrupt the encompassing gene due to the presence of transcription termination sites in the LTRs, which leads to negative selection of such integration events [39]. Contrarily, reverse oriented proviruses may even be beneficial by protecting from newly infecting retroviruses by antisense RNA mechanisms [19] and by contributing regulatory elements such as LTR promoters that can modulate gene expression in *cis*, as shown before [7, 14].

### HML-10 exerts differential LTR promoter activity in tumor cell lines

**Fig. 3:**
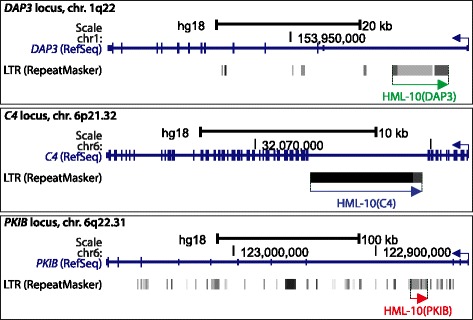
Genomic organization of the HML-10(DAP3), HML-10(C4) and HML-10(PKIB) proviruses (from *top* to *bottom*). The grey rectangles in the LTR (RepeatMasker) track shows all annotated HERV elements including the indicated HML-10 proviruses. Images were retrieved and modified from the UCSC Genome Browser [68]

To further investigate the potential of HML-10 in generating *cis*-regulatory transcripts, we determined LTR promoter activities of three complete proviruses located in reverse orientation within introns of host genes (Fig. 3). These were elements nos. 3, 22 and 25, within the *DAP3*, *C4* and *PKIB* (Protein kinase inhibitor beta) genes, respectively (Table 1). The *PKIB* gene harbors numerous other intronic HERV sequences not belonging to the HML-10 family that together with other REs constitute over 50% of its genomic sequence. Three additional HML-10 proviruses are located outside of genes, elements nos. 1, 20 and 68, and one found in an intron of the zinc finger protein gene *ZNF578*, no. 58, lacks the 5′LTR. We focused on the three complete and intronic proviruses, referred to as HML-10(DAP3), HML-10(C4) and HML-10(PKIB), that comprise six LTRs for promoter analysis, since these could potentially generate *cis*-regulatory transcripts. We preferred proviruses over solitary LTRs since proviral LTRs of the related HML-2 and HERV-W families have been shown to be stronger promoters than the respective solitary LTRs [8, 15]. We also found that the two LTRs of each HML-10 provirus clustered in the neighbor-joining tree (Fig. 2). Thus, despite their high sequence similarities these LTRs have resisted homologous recombination, suggesting their functional importance. HML-10 provirus RNA has been detected in various human tissues and cell lines by microarray analyses [22–27, 40–43] that, however, lack the information whether transcription is initiated in the 5′LTR or upstream of the provirus.

**Fig. 4:**
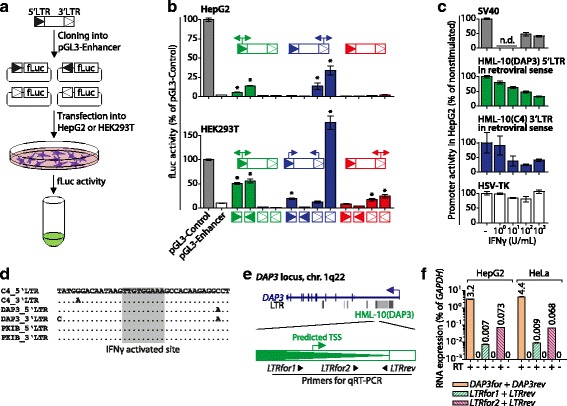
Promoter activities of HML-10 LTRs. **a** LTRs of the HML-10(DAP3), HML-10(C4) and HML-10(PKIB) proviruses were cloned in both orientations into the promoter-free pGL3-Enhancer vector and transfected into HepG2 or HEK293T cells. Firefly luciferase (fLuc) activities were determined 24 h after transfection **b** Promoter activities expressed as fLuc activity normalized to renilla luciferase (rLuc) activity of the co-transfected pGL4.74 vector in the indicated cell lines. The pGL3-Control vector bearing the SV40 promoter (*grey bars*) served as positive and empty pGL3-Enhancer (*white bars*) as negative control. Promoter activities were normalized to pGL3-Control set to 100%. The bars show mean ± SEM of three independent experiments in duplicates. *P-value ≤ 0.05, Student’s *t*-Test compared to pGL3-Enhancer. **c** For HepG2 cells the effect of IFNγ stimulation on two selected LTRs as well as the SV40 and HSV-TK promoters is shown. LTR and SV40 activity is expressed as fLuc normalized to rLuc signals, HSV-TK activity is expressed as rLuc activity only. The *bars* show mean ± SEM of at least three independent experiments and were normalized to unstimulated (-) cells set to 100%. n.d., not determined. **d** Identification of a conserved IFNγ activated site (GAS) of the consensus sequence 5′-TTNCNNNAA-3′ [45]. **e** Locations of primers used to detect transcripts originating from the 5′LTR of HML-10(DAP3). The predicted TSS was identified as described in the text and Additional file 1: Figure S1. **f** Detection of *DAP3* mRNA and HML-10(DAP3) transcripts in HepG2 and HeLa cells by qRT-PCR. cDNA samples prepared without reverse transcriptase (RT) for the indicated primer pairs, but with RT for *GAPDH*, served as controls. Values are normalized to *GAPDH* mRNA levels. *Bars* show mean ± SD of two measurements. In most cases, the SD is too small to be visible

To assess their promoter activities, we cloned the LTRs of HML-10(DAP3), HML-10(C4) and HML-10(PKIB) into the promoter-free pGL3-Enhancer luciferase reporter vector, as described [19] (Fig. 4a). As HERV LTRs can be bidirectional promoters [5, 17, 44], we also included the retroviral antisense orientation for each of the six LTRs. LTR promoter activity of HML-10(C4) has been demonstrated before with reporter assays in the human hepatocellular carcinoma cell line HepG2 and in COS7 monkey kidney cells [19]. Additionally, HML-10 *pol* transcripts have been identified in human hepatocellular carcinoma cells and in human embryonic kidney HEK293 cells by microarray analysis [26] (Table 2). Based on these findings, we transfected our pGL3-Enhancer constructs into HepG2 and HEK293T cells (HEK293 expressing SV40 virus T-antigen) to measure their promoter activities (Fig. 4b). The pGL3-Control vector bearing the SV40 promoter served as positive control and empty, promoter-free pGL3-Enhancer as negative control. HML-10(C4) showed significant transcriptional activity exclusively in the 3′LTR in HepG2 in both retroviral sense and antisense orientations. This is in accordance with a previous study that has demonstrated promoter activity in the 3′LTR, but not in the 5′LTR of this provirus in the same cell line [19]. In HEK293T, we found transcription from the 5′LTR in retroviral sense orientation and from the 3′LTR in retroviral antisense orientation. HML-10(DAP3) exerted bidirectional promoter activity in its 5′LTR in both cell lines, whereas HML-10(PKIB) showed bidirectional promoter activity in its 3′LTR, but in HEK293T only. Therefore, all three investigated proviruses showed transcriptional activity in at least one of their LTRs, with cell type-specific strength and orientation (Fig. 4b). While LTR promoter activity in retroviral antisense orientation was unlikely to primarily affect gene regulation, all three HERVs exerted promoter activity in retroviral sense orientation in one of their LTRs, which is antisense relative to the respective encompassing gene. Thus, the proviruses have the potential for antisense-mediated regulation of the encompassing *DAP3*, *C4* and *PKIB* genes in *cis* in a cell type-specific manner.

**Table 2:** Detection of HML-10(DAP3) *pol* transcripts by previously reported microarray studies [79]

Tissue/cell type	HML-10(DAP3) RNA expressed	HML-10(DAP3) RNA not expressed	HML-10(DAP3) RNA variably expressed
Normal tissue and non-tumor cell lines (*n* = 23)	Cervix [22], epidermal keratinocytes (HaCaT) [26], thyroid [22], umbilical vein endothelial cells (HUVEC) [40], uterus [22] (5/23)	Blood [22, 23], breast [25], colon [22], heart [22], liver [22], lung [22], mamma [22, 23], neural stem cells (HNSC.100) [40], ovary [22], placenta [22], prostate [22], rectum [22], skeletal muscle [22], stomach [22], testes [22] (15/23)	Brain [22, 24], kidney [22, 27], skin [22, 41] (3/23)
Primary tumors and tumor cell lines (*n* = 13)	CAKI (renal carcinoma) [26], GliNS1 (neural tumor stem line) [40], HEK293 (embryonic kidney) [26], HeLa (cervix adenocarcinoma) [26], HuH-7 (hepatocellular carcinoma) [26], MIA PaCa-2 (pancreatic carcinoma) [26], SK-N-MC (neuroblastoma) [42, 43], SK-N-SH (neuroblastoma) [42], T47D (ductal breast epithelial carcinoma) [26], U-251MG (glioblastoma) [42] (10/13)	Breast cancer [25], U-138MG (glioblastoma) [42] (2/13)	Renal cell carcinoma [27] (1/13)

Numbers in parentheses indicate how many of the analyzed tissue/cell types show the respective expression status of HML-10(DAP3) RNA

Promoter activity of the HML-10(C4) 3′LTR has been reported previously to be suppressed by IFNγ in HepG2 [19], which we reproduced (Fig. 4c). Likewise, the 5′LTR promoter of HML-10(DAP3) in retroviral sense orientation (antisense relative to the *DAP3* gene) was dose-dependently suppressed by IFNγ. We speculate this to be mediated by an IFNγ activated site (GAS) matching the consensus motif 5′-TTNCNNNAA-3′, a putative binding site for STAT1 homodimers that form during IFNγ signaling [45]. This motif is present in all analyzed LTRs (Fig. 4d) as well as the SV40 promoter (data not shown). The latter is known to be inhibited by IFNγ [46] and served as positive control for IFNγ-mediated suppression (Fig. 4c). In contrast, the herpes simplex virus thymidine kinase (HSV-TK) promoter that was used to normalize promoter activities was unaffected by IFNγ (Fig. 4c). The GAS motif is highly conserved among proviral HML-10 LTRs in the human genome (Fig. 4d) and the solitary LTRs (data not shown), which supports its functional relevance. Hence, IFNγ-mediated promoter suppression is likely a general feature of HML-10 LTRs, in line with the known antiviral activity of interferons [19]. This is of particular interest for possible HML-10-mediated negative regulation of the encompassing genes, since mRNA expression of *C4* and *DAP3* is known to be induced by IFNγ [20, 47] and *DAP3* is implicated in IFNγ-dependent apoptosis [20].

Based on our promoter activity studies, HML-10(DAP3) was the most interesting candidate for further investigation, since its 5′LTR is the only one investigated that promoted transcription in the retroviral sense orientation, which is antisense to *DAP3*, in both cell lines (Fig. 4b). The involvement of an HML-10(DAP3)-primed transcript in regulating the encompassing gene is suggested by the fact that *DAP3* expression is induced [20], whereas the LTR promoter is suppressed by IFNγ (Fig. 4c). In addition, HML-10(DAP3) RNA has been detected previously in various human cancer-derived cell lines but not in most healthy tissues (Table 2). This indicates a possible role in the regulation of *DAP3* gene expression in cancer cells and some distinct tissues, including cervix, thyroid and uterus as well as epidermal keratinocytes and umbilical vein endothelial cells. Our promoter activity studies indicated that transcription of HML-10(DAP3) originated from the 5′LTR (Fig. 4b). For further proof, we determined the most likely TSS within this promoter. Since LTR-dependent transcription relies on host RNA polymerase (RNA pol) II [5, 48] we sought to identify the two integral core elements of this promoter, Initiator (Inr) elements and TATA boxes [49]. The TSS within LTRs of the related HML-2 family has been identified previously within an Inr element with a TATA box about 10 bp upstream of the Inr [50]. We identified a similar configuration a single time in the HML-10(DAP3) 5′LTR in retroviral sense orientation, an Inr element 11 bp downstream of a TATA box (Additional file 3: Figure S2). This Inr sequence contained the most likely TSS. We also identified a downstream promoter element (DPE) matching the consensus 5′-RGWYVT-3′ sequence [49], a putative binding site for the transcription factor TFIID of the RNA pol II core promoter, at nucleotide position +19 relative to the putative TSS. To get experimental proof that HML-10(DAP3) transcription is initiated within this putative TSS, we performed quantitative real-time PCR (qRT-PCR) measurements in HepG2 cells with a reverse primer located downstream of the TSS (*LTRrev*) and two different forward primers, one located upstream (*LTRfor1*) and one downstream (*LTRfor2*) of the TSS (Fig. 4e and Additional file 3: Figure S2). If transcription was initiated from the TSS, we would expect higher expression measured using *LTRfor2* + *LTRrev* than with *LTRfor1* + *LTRrev* primer combinations. This was indeed the case, whereby weak signals seen with *LTRfor1* + *LTRrev* likely resulted from amplification of the intron of the *DAP3* pre-mRNA (Fig. 4f). To avoid false signals from genomic DNA for these lowly abundant transcripts, we subjected the RNA preparations to DNase treatment prior to reverse transcription and included control samples without reverse transcriptase that did not result in detectable amplification. We thus verified expression of the HML-10(DAP3) RNA that is present at about 40-fold lower levels than the *DAP3* mRNA, and provide further evidence that it originates from the 5′LTR around the predicted TSS. These findings confirmed the weak but significant transcription of this LTR in the promoter activity studies in retroviral sense orientation in the same cell line (Fig. 4b). Our findings are in agreement with reported microarray data that demonstrated expression of the retroviral transcript in various cell lines, which extends into the *pol* gene of the HML-10(DAP3) provirus (Table 2). However, although the primer combinations were designed to only amplify the HML-10(DAP3) sequence, as judged by *in silico* PCR analysis, we cannot completely rule out that transcripts of other potentially active HML-10 elements were co-amplified.

The pro-apoptotic effect of *DAP3* has been described previously in HeLa cells [20] in which HML-10(DAP3) RNA has been identified by microarray analysis (Table 2). Accordingly, we detected HML-10-primed transcripts by qRT-PCR in HeLa where it was present at comparable levels as in HepG2 (Fig. 4f). We therefore selected HeLa cells to determine the functional relevance of HML-10(DAP3) RNA on the expression of *DAP3*.

### Inactivation of HML-10(DAP3) RNA induces *DAP3* expression and apoptosis in HeLa cells

Having confirmed the presence of HML-10(DAP3) RNA in HeLa cells and its likely origin within the proviral 5′LTR, we sought to determine its function within the cell. We expected the retroviral RNA to suppress *DAP3* gene expression in *cis* similar to the previously described LTR-primed regulatory transcripts [7, 14]. To determine its potential regulatory function we aimed at inactivating the retroviral RNA by means of sequence-specific ASOs. We opted for ASOs rather than siRNAs that are both known to be active in the nucleus [51, 52], the common site of action of LTR-primed transcripts [5], as siRNAs may directly influence DAP3 expression levels through the passenger strand that would be antisense to the DAP3 pre-mRNA. ASO-mediated inactivation of the HML-10(DAP3) RNA was expected to activate *DAP3* gene expression.

**Fig. 5:**
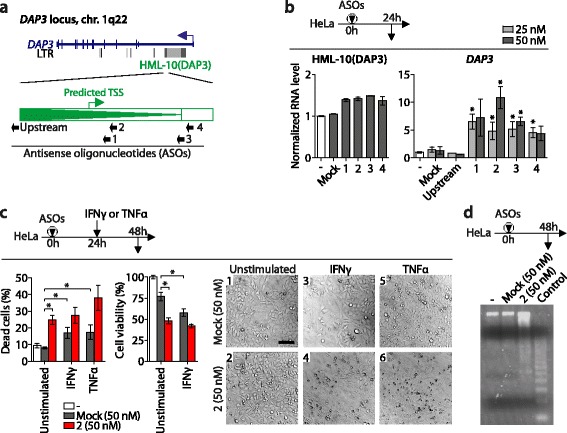
Inactivating the HML-10(DAP3) RNA induces *DAP3* expression and apoptosis in HeLa cells. **a** Target regions of sequence-specific ASOs are indicated. ASOs 1-4 are in antisense orientation to the retroviral transcript and in sense orientation to the *DAP3* transcript. The ASO designated as Upstream served as control. **b** Cells were transfected with 25 or 50 nM of the indicated ASOs. At 24 h after transfection, expression levels of HML-10(DAP3) (*left*) and *DAP3* mRNA (*right*) were determined by qRT-PCR. *Bars* show mean ± SEM of three independent experiments. RNA levels were normalized to *GAPDH* and levels of non-transfected cells were set to 1. *P-value ≤ 0.05, Student’s *t*-Test against Mock. **c** Cells were transfected with the indicated ASOs at 50 nM, after 24 h stimulated with 1000 U/mL IFNγ or 100 ng/mL TNFα, or left unstimulated. After additional 24 h, *Trypan Blue* exclusion as indicator of dead cells (*left*), MTS cell viability assays (*center*) or light microscopic analysis (*right*) was performed. *Bars* show mean ± SEM of three independent experiments in duplicates. *P-value ≤ 0.05, Student’s *t*-Test. The *scale bar* in light microscopy panel 1 is 100 μm. **d** Cells were transfected with the indicated ASOs at 50 nM. At 48 h after transfection, genomic DNA of these cells was prepared with the Apoptotic DNA Ladder Kit (Roche). The control DNA is from apoptotic U937 cells provided with the kit

We designed four ASOs downstream of the putative TSS, ASOs 1–4, to counteract the retroviral RNA (Fig. 5a). At 24 h after transfecting the ASOs at 25 or 50 nM into HeLa cells, we determined HML-10(DAP3) and *DAP3* expression at the RNA level by qRT-PCR. Transfecting the ASOs caused an increase in *DAP3* mRNA levels, as expected, but not a decrease in HML-10(DAP3) RNA (Fig. 5b). These observations likely indicate that the ASOs blocked association of *DAP3* pre-mRNA with the retroviral RNA, but did not significantly mediate cleavage of the latter. Although RNase H1/H2-dependent hybrid-specific RNA degradation has been reported to be induced by ASOs [51, 53], cleavage efficiency is largely sequence-dependent and the HML-10(DAP3) RNA might resist degradation. For these reasons, measuring *DAP3* mRNA levels was the only feasible way to assess the impact of inactivating the retroviral RNA. Transfecting ASOs 1–4 resulted in increased *DAP3* mRNA levels with varying efficiencies (Fig. 5b). When used at 25 nM, ASOs 1–4 increased *DAP3* mRNA levels about 5-fold as compared to non-transfected control cells. The most efficient ASO 2 exerted a dose-dependent increase of the *DAP3* mRNA up to 10-fold at 50 nM. Both control ASOs, one with a random sequence (Mock) and one immediately upstream of the 5′LTR, did not significantly change *DAP3* expression levels, demonstrating a sequence-dependent effect and that the HML-10(DAP3) RNA originates from the 5′LTR. Although ASOs 1–4 were designed to only map to the *DAP3* locus, we consider the possibility that HML-10 RNA species transcribed at other loci that may act *in trans* on *DAP3* expression may have been inactivated by these ASOs as well. Overall, the use of ASOs to counteract HML-10-primed transcripts confirmed their negative impact on *DAP3* mRNA expression levels.

DAP3 is an adapter protein that links the intracellular portion of DRs to the Fas-Associated Death Domain (FADD) in the DR pathway of extrinsic apoptosis [21]. Consequently, we expected the HML-10(DAP3) RNA to suppress apoptosis via this pathway. Overexpressing *DAP3* has been shown to induce apoptosis in HeLa cells [20]. We wondered whether upregulation of *DAP3* by the most effective ASO 2 at 50 nM (Fig. 5b) was sufficient to cause apoptosis. To this end, we compared the effect of ASO 2 with known apoptosis-inducing stimuli, tumor necrosis factor-alpha (TNFα) and IFNγ, on HeLa cells. Both cytokines significantly induced cell death associated with diminished cell viability, as well as cell rounding characteristic of apoptosis (Fig. 5c). Likewise, HeLa cells transfected with ASO 2 showed similar signs of apoptosis but not those transfected with Mock ASO. The fraction of dead cells was significantly higher for ASO 2-transfected cells when compared to Mock-transfected cells (24.8% vs. 8.0%, *P* = 10^−4^), and cell viability was lower (47.9% vs. 76.8% relative to non-transfected cells, *P* = 10^−4^). In addition, transfection of ASO 2, but not of Mock, induced features of apoptosis, such as detachment from the tissue culture dish, rounding and shrinking (Fig. 5c, light microscopy panels 1 and 2). This was supported by another test for apoptosis, genomic DNA fragmentation, that occurred upon transfection of ASO 2 (Fig. 5d). These findings provided evidence that ASO 2-mediated induction of *DAP3* mRNA led to increased expression of DAP3 protein that is required for apoptosis and DNA fragmentation. Thus, inactivating HML-10(DAP3) RNA increased *DAP3* expression sufficiently to induce apoptosis, which demonstrates the functional relevance of this retroviral transcript.

In parallel, we assessed whether inactivating HML-10(DAP3) RNA also increased the susceptibility to apoptosis by TNFα. We expected this since TNFα stimulates extrinsic apoptosis via the DR pathway that involves DAP3 [21]. Thus, inactivation of HML-10(DAP3) RNA with resulting *DAP3* overexpression and TNFα stimulation may promote apoptosis synergistically. Indeed, ASO 2-transfected HeLa cells that were additionally stimulated with TNFα exhibited increased signs of apoptosis compared to unstimulated ASO 2-transfected cells (Fig. 5c, light microscopy panels 2 and 6), and contained a larger fraction of dead cells (38.1% vs. 24.8%), albeit without statistical significance (Fig. 5c). Stimulating ASO 2-transfected cells with IFNγ had less pronounced effects on the fraction of dead cells and viability (Fig. 5c), which may be because IFNγ induces apoptosis independent of DR signaling. Conclusively, we showed that *DAP3* expression is negatively regulated by the HML-10(DAP3) RNA to an extent that apoptosis is inhibited in HeLa cells.

## Discussion

Here we have characterized the previously little described HML-10 family of endogenous retroviruses in the human genome and studied its potential in regulating host gene expression. We found that the infectious progenitor of HML-10 invaded the ancestral genome of OWMs about 35 Mya (Fig. 1b). With 70 identified elements, HML-10 is a relatively small HERV family when compared, for instance, to the intensely investigated HML-2 that constitutes about 2500 sequences in the human genome [4]. It is known that HERVs, after *de novo* integration, may increase in number due to chromosomal duplication events [4]. However, sequence comparisons of the TSDs (Table 1) and the flanking regions (Additional file 1: Figure S1) indicated that only one of the 70 identified HML-10 elements is the result of a chromosomal duplication, whereas the other 69 elements most likely arose by independent retroviral integrations. We found an unusually high abundance of HML-10 within introns of host genes when compared to other HERV sequences including those of phylogenetically related HML families (Fig. 1e), indicating that this family in particular has been evolutionary co-opted for gene regulatory functions. Since LTR promoter activity of the provirus in the *C4* gene has been demonstrated previously [19], we hypothesized HML-10 to express LTR-primed regulatory transcripts in *cis* similar to recently reported HML-2 [14] and HERV-E [7] LTRs.

To assess their potential in expressing such regulatory RNAs, LTRs of three selected, intron-located HML-10 proviruses were subjected to promoter activity studies in HepG2 and HEK293T cells (Fig. 4b). Interestingly, both strength and orientation of LTR transcription differed substantially between the cell lines. Based on the promoter activity studies, all three investigated HML-10 proviruses had the potential to negatively regulate their encompassing genes by priming antisense RNAs. The HML-10(DAP3) provirus located in the *DAP3* gene showed LTR promoter activity in retroviral sense orientation (antisense relative to *DAP3*) in both cell lines and was therefore selected for further analysis (Fig. 4b). DAP3 is a signaling protein involved in the DR pathway of extrinsic apoptosis that induces apoptosis when overexpressed [20, 21]. Promoter activity of the HML-10(DAP3) 5′LTR in retroviral sense orientation (antisense relative to the *DAP3* gene) was suppressed by IFNγ, as reported previously for the HML-10 provirus in the *C4* gene [19] (Fig. 4c). This might, at least partially, explain how *DAP3* gene expression is induced by IFNγ [20]. In HeLa cells, we found that counteracting the retroviral transcript by sequence-specific ASOs led to an increase of *DAP3* expression levels sufficient to induce apoptosis (Fig. 5b,c). Two control ASOs, one targeting a region upstream of and one with a randomized sequence, did neither induce *DAP3* mRNA expression nor apoptosis, verifying that the ASO transfection procedure itself did not exert any non-specific effects on these two read-outs. Thus, the HML-10(DAP3) RNA suppressed apoptosis in HeLa. HML-10 transcripts originating from other loci may have been inactivated by the ASOs as well and consequently might also contribute to the reduction of *DAP3* expression *in trans*. ASO-mediated inactivation confirmed that the HML-10-primed transcripts, despite being about 60-fold weaker expressed than the DAP3 mRNA in this cell line (Fig. 4f), had a substantial impact on *DAP3* expression levels. Indeed, regulatory non-coding RNAs are often weakly expressed [54] and capable of substantially down-regulating gene expression even if 10-100 fold less abundant than their respective mRNA [55]. Among the mechanisms that have been proposed for this kind of gene regulation is the induction of repressive epigenetic modifications that lead to heterochromatin formation, or transcriptional collision of opposing RNA polymerases [54]. It has been shown previously that preventing association between weakly expressed regulatory RNAs and their corresponding mRNA (as opposed to degradation of the regulatory RNA) is sufficient to substantially induce mRNA expression [55], which might explain why we did not observe ASO-mediated degradation of HML-10 RNA but nevertheless an increase in *DAP3* mRNA expression levels (Fig. 5b).

Promoter activity studies (Fig. 4b), qRT-PCR experiments (Fig. 4f), and the fact that the ASO immediately upstream of the LTR did not affect *DAP3* expression levels (Fig. 5b) provided evidence that the retroviral RNA originates from the proviral 5′LTR. We determined the most likely TSS within this LTR by *in silico* sequence analysis (Additional file 3: Figure S2). Attempts to experimentally verify this TSS by 5′RACE-PCR as described previously [14] were not successful, as orientation-specific cDNA synthesis did not yield sufficient starting material for subsequent PCR reactions (see Methods section for details). Insufficient orientation-specific cDNA synthesis may have been due to the low abundance of the HML-10(DAP3) RNA as seen by qRT-PCR (Fig. 4f) and is a known issue with rare transcripts [56]. Thus, the actual TSS of the retroviral transcript may differ from the predicted one but our experiments provide evidence that it is located between the target regions of ASO upstream and ASO 1 (Fig. 5a). Our findings indirectly confirmed the expression of HML-10(DAP3) RNA in HeLa cells, which was supported by reported microarray experiments (Table 2). Further direct proof could be obtained by sequencing cDNA clones and identifying genomic markers that are unique to the HML-10(DAP3) copy, such as the *AluSp* or *MER11B* repeats that are integrated into this provirus (Table 1).

Suppression of apoptosis, as mediated by the HML-10(DAP3) RNA in HeLa cells, is a general hallmark of cancer cells [57]. Thus, retroviral transcripts may contribute to the malignant cellular phenotype of this cell line by counteracting *DAP3* expression, and thereby suppressing apoptosis. Aberrant expression levels of *DAP3* have been suggested to play a role in some cases of malignant disease [58–63]. The data shown here indicate that most of the HML-10 LTRs are even weaker expressed than the one analyzed. We hypothesize that the LTRs, which are normally strong promoters in infectious retroviruses, have been silenced by mutation during evolution. Thus, they likely play a limited role in cancer promotion.

The data presented in Fig. 4b-c suggests that the expression of LTR-primed transcripts varies substantially in intensity and direction depending on the cell type as well as the action of cytokines. Moreover, despite its weak expression at about 60-fold lower levels than *DAP3* mRNA (Fig. 4f) the HML-10-primed RNA had a strong impact on *DAP3* gene regulation (Fig. 5b). Consistent contributions of this and other HERV-primed RNAs to various tissues or tumors may therefore be hard to identify. However, the presence of HML-10(DAP3) RNA in many tumor cell lines and the absence in most healthy tissues (Table 2) suggest that its upregulation may be a relevant feature in some human cancer diseases. This is in line with the observation that transcriptional activation of HERVs and other REs by epigenetic DNA demethylation is a frequent characteristic of malignant cells [64–66].

## Conclusions

This work provides experimental support for recent evidence that HERVs and other REs play a role in gene regulation and cellular processes relevant to mammalian tumor cell formation. In the case presented here, transcripts of the previously little described HML-10 family suppressed the pro-apoptotic *DAP3* gene and consequently, apoptosis in HeLa cells. Therefore, we could verify a direct link between HERV expression and cellular phenotype in this cell line. A potential role of these LTRs in promoting a malignant phenotype possibly by inducing resistance to apoptosis as described here in other cell lines or tissues requires further investigation.

## Methods

### Identification of HML-10 elements in the human genome

The Table Browser function [67] of the UCSC Genome Browser [68] was used to identify HML-10 elements in the human genome. We queried the Repbase sequence of HML-10 LTRs, *LTR14* [69], in the RepeatMasker track [70] of the GRCh38/hg38 human genome assembly [1]. This search yielded 86 hits. Manual inspection of these hits revealed the 70 unique HML-10 elements listed in Table 1.

### Estimation of the evolutionary age of HML-10 proviruses

For each of the six complete HML-10 proviruses (elements Nos. 1, 3, 20, 22, 25 and 68 in Table 1), both LTR sequences (5′ and 3′LTRs) were aligned with Clustal X 2.0 [71]. The evolutionary age of each provirus was calculated from the number of mutations between both LTRs by applying an estimated nucleotide substitution rate of 2.28 per site and year × 10^−9^ as described [28].

### Construction of phylogenetic neighbor-joining trees

The *pol* sequences of HML-10 and other betaretroviruses were retrieved from published literature [18, 72]. The fasta protein sequences can be found in Additional file 4. Sequences were aligned with Clustal X 2.0 [71] using standard parameters of the *Multiple Alignment Mode*. The neighbor-joining tree was visualized with TreeView 1.6.6 [73]. The phylogenetic tree of HML-10 LTR nucleotide sequences and those of the flanking sequences shown in Additional file 1: Figure S1 were constructed similarly. All nucleotide sequences were retrieved from the UCSC Genome Browser [68] and the current release of the human genome, GRCh38/hg38 [1].

### Identification of target site duplications

The sequences immediately up- and downstream of RepeatMasker-annotated HML-10 elements (Table 1) were searched for homologous sequences in the retroviral sense orientation. Homologous sequences of at least 5 bp were defined as TSDs, allowing for one (5 bp TSDs) or two (6 bp TSDs) nucleotide mismatches.

### Location of HERV sequences relative to human genes

The fractions of intragenic HERV sequences were determined with the UCSC Table Browser [67] using the GRCh38/hg38 human genome assembly [1]. HERV elements were identified as described below in this paragraph within the RepeatMasker track [70]. The output of these searches was used to generate custom tracks covering the sequences of the respective HERV families. Using the *intersection* function, the overlap of HERV sequences with a custom track representing full-length RefSeq genes was determined, yielding the following values (displayed as: HERV family, Repbase annotation, sequence covered, sequence intersected with RefSeq genes): HML-1, *LTR14A* / *LTR14B* / *LTR14C*, 274,910 bp, 66,940 bp (24.35%); HML-2, *LTR5A* / *LTR5B*, 595,281 bp, 171,219 bp (28.76%); HML-3, *MER9B* / *MER9a1* / *MER9a2* / *MER9a3*, 568,179 bp, 151,429 bp (26.65%); HML-4, *LTR13* / *LTR13A*, 545,702 bp, 200,556 bp (26.75%); HML-5, *LTR22* / *LTR22A* / *LTR22B* / *LTR22B1* / *LTR22B2* / *LTR22C* / *LTR22C0* / *LTR22C2* / *LTR22E*, 396,533 bp, 105,855 bp (26.70%); HML-6, *LTR3* / *LTR3A* / *LTR3B*, 130,701 bp, 37,058 bp (28.35%); HML-7, *MER11D*, 194,536 bp, 60,756 bp (31.23%); HML-8, *MER11A* / *MER11B* / *MER11C*, 2,222,448 bp, 656,281 bp (29.53%); HML-10, *LTR14*, 40,556 bp, 19,443 bp (47.94%); HERV-W, *LTR17*, 482,257 bp, 134,803 bp (27.95%). All RepeatMasker-annotated HERV elements covered 266,970,452 bp, of which 75,967,800 bp (28.46%) intersected with RefSeq genes. The fraction of the total genome (3,088,269,808 bp) accounted for RefSeq genes was 1,320,982,363 bp (44.97%).

### Cell lines and culture conditions

HeLa (ATCC CCL-2), HepG2 (ATCC HB-8065) and HEK293T cell lines were cultivated in complete growth medium; Dulbecco’s Modified Eagle’s Medium (DMEM) (Invitrogen, Carlsbad, CA, USA) supplemented with 10% heat-inactivated fetal calf serum (Invitrogen) and 100 U/mL penicillin, 100 μg/mL streptomycin and 0.25 μg/mL amphotericin (Antibiotic-Antimycotic by Invitrogen). Cells were incubated at 37 °C with 5% CO_2_. Subcultivation ratios ranged between 1:2 and 1:10.

### Primers

All primers were synthesized by Microsynth AG, Balgach, Switzerland. Primer sequences are listed in Additional file 5. Primer sequences were designed such that they only amplified the desired regions, as verified by the *in silico* PCR analysis tool of UCSC on https://genome.ucsc.edu/cgi-bin/hgPcr/.

### Construction of pGL3-Enhancer luciferase reporter vectors

LTRs of HML-10(C4), HML-10(DAP3) and HML-10(PKIB) were amplified by standard PCR from genomic DNA of the QBL cell line (No. 4070713) obtained from the Health Protection Agency Culture Collections (ECACC, Salisbury, UK), using primer pairs with a HindIII or XhoI cleavage site at their 5′ ends. HML-10(C4) primer pairs: 5′LTR(s), *C4_5LTRforHindIII* + *C4_5LTRrevXhoI*; 5′LTR(as), *C4_5LTRforXhoI* + *C4_5LTRrevHindIII*; 3′LTR(s), *C4_3LTRforHindIII* + *C4_3LTRrevXhoI*; 3′LTR(as), *C4_3LTRforXhoI* + *C4_3LTRrevHindIII*. HML-10(DAP3) primer pairs: 5′LTR(s), *DAP3_5LTRforHindIII* + *DAP3_5LTRrevXhoI*; 5′LTR(as), *DAP3_5LTRforXhoI* + *DAP3_5LTRrevHindIII*; 3′LTR(s), *DAP3_3LTRforHindIII* + *DAP3_3LTRrevXhoI*; 3′LTR(as), *DAP3_3LTRforXhoI* + *DAP3_3LTRrevHindIII*. HML-10(PKIB) primer pairs: 5′LTR(s), *PKIB_5LTRforHindIII* + *PKIB_5LTRrevXhoI*; 5′LTR(as), *PKIB_5LTRforXhoI* + *PKIB_5LTRrevHindIII*; 3′LTR(s), *PKIB_3LTRforHindIII* + *PKIB_3LTRrevXhoI*; 3′LTR(as), *PKIB_3LTRforXhoI* + *PKIB_3LTRrevHindIII*). Cycling conditions were 10 min. 95 °C; (30 s. 95 °C, 30 s. 60 °C, 30 s. 72 °C) × 40; 7 min. 72 °C. LTRs were cloned into the pGL3-Enhancer vector (Promega, Madison, WI, USA), containing the fLuc gene as reporter, after digestion with HindIII and XhoI (New England Biolabs, Ipswich, MA, USA) and phosphatase treatment. Vectors were ligated with T4 DNA Ligase (New England Biolabs). All vector constructs were heat-shock-transformed into competent *E. coli* JM109 (Promega). Positive colonies were detected by ampicillin resistance on selective agar plates. Selected clones were grown in ampicillin-containing LB medium and plasmid DNA was isolated with the QIAamp Plasmid DNA Mini Kit (Qiagen, Hilden, Germany). Plasmid DNAs were screened for correct inserts by restriction enzyme digestions using appropriate enzyme combinations and subsequent agarose gel electrophoresis as well as by capillary sequencing (Microsynth, Balgach, Switzerland).

### Determination of LTR promoter activities

Freshly passaged HepG2 or HEK293T cells were seeded into 24-well tissue culture plates (4x10^4^ cells per well in complete growth medium) and cultivated overnight to ~80% confluence. Cells were transfected with 50 ng/well of pGL3-Enhancer constructs, empty pGL3-Enhancer, or pGL3-Control, 4 ng/well of pGL4.74 vector for normalization (Promega) and 346 ng/well of unrelated carrier DNA using DreamFect Gold transfection reagent (OZ Biosciences, Marseille, France) following the manufacturer’s recommendations. Vector pGL4.74 contains the renilla luciferase (rLuc) gene under control of the herpes simplex virus thymidine kinase (HSV-TK) promoter. Medium was replaced with fresh prewarmed complete growth medium 6 h post-transfection. At 24 h post-transfection, medium was aspirated, cells were rinsed with prewarmed PBS, lysed, and fLuc and rLuc activities in each sample were determined with the Dual-Glo Luciferase Assay System (Promega) in a Sirius Luminometer (Berthold Detection Systems, Pforzheim, Germany). fLuc activities were normalized to rLuc activities for each sample. To assess the effect of IFNγ stimulation on promoter activities, selected pGL3-Enhancer constructs were transfected into HepG2 cells as above and were stimulated with different amounts of recombinant human IFNγ (PeproTech, Rocky Hill, NJ, USA) by addition to the growth medium immediately after medium change 6 h post-transfection. fLuc activities were determined 30 h post-transfection.

### Inactivation of HML-10(DAP3) RNA with ASOs

The ASOs were 25-mer DNA molecules with phosphorothioate bonds at the flanking three nucleotides on both sides to confer exonuclease resistance. ASOs for inactivating the HML-10(DAP3) RNA were designed to be complementary to regions within the 5′LTR or the proviral body downstream of the predicted TSS. We used only sequences that uniquely mapped to their respective target region and nowhere else in the human genome. A Mock ASO with a randomized sequence and one complementary to a region shortly upstream of the 5′LTR were used as negative controls. ASOs were purchased from Microsynth. Their sequences are listed in Additional file 6.

### qRT-PCR

Freshly passaged HepG2 or HeLa cells were seeded in 96-well plates (10^4^ cells per well in complete growth medium) and grown overnight to ~80% confluence. Cells were transfected with 25 or 50 nM of the indicated ASOs using DreamFect Gold transfection reagent (OZ Biosciences) according to the manufacturer’s recommendations. Medium was replaced with fresh prewarmed complete growth medium 6 h post-transfection. At 24 h post-transfection, total RNA was extracted using the QIAamp RNA Blood Mini Kit (Qiagen), including an on-column DNA digestion step with the RNase-free DNase Set (Qiagen). First strand cDNA was synthesized using the High Capacity cDNA Reverse Transcription Kit (Applied Biosystems, Foster City, CA, USA) with random hexamer primers. qRT-PCR was performed using the TaqMan Universal PCR Master Mix (Applied Biosystems) with the addition of 1:10000 (v/v) SYBR Green I (Sigma-Aldrich, St. Louis, MO, USA) and primers specific for *DAP3* (*DAP3for* + *DAP3rev*) or *GAPDH* (*GAPDHfor* + *GAPDHrev*) mRNAs, HML-10(DAP3) RNA (*LTRfor2* + *LTRrev*) or *LTRfor1* + *LTRrev* as control reaction. Cycling conditions were 2 min. 50 °C; 10 min. 95 °C; (15 s. 95 °C, 1 min. 58 °C) × 65. Specificity of the PCR reactions was assessed by checking for correct amplicon lengths and amplification artifacts by agarose gel electrophoresis. All shown RNA levels were calculated by relative quantification (double delta Ct method) using *GAPDH* as reference, with primer efficiencies calculated from serial dilutions of HepG2 cDNA samples. Control samples without addition of reverse transcriptase gave no amplification signals.

### Strand-specific cDNA synthesis

A number of 10^6^ freshly passaged HepG2 or HeLa cells were seeded into wells of 6-well plates and grown overnight to ~80% confluence. Total RNA was extracted using the QIAamp RNA Blood Mini Kit (Qiagen). First strand cDNA was synthesized using either the Reverse Transcriptase of the High Capacity cDNA Reverse Transcription Kit (Applied Biosystems), or the Thermoscript Reverse Transcriptase (Invitrogen) with primers specific for the HML-10(DAP3) transcript (Additional file 5). Different incubation times and temperatures (ranging from 25 to 60 °C) were evaluated. To assess reverse transcription efficiencies, qRT-PCR was done using TaqMan Universal PCR Master Mix (Applied Biosystems) with the addition of 1:10000 (v/v) SYBR Green I (Sigma-Aldrich) and primers *LTRfor2* + *LTRrev*. Cycling conditions were 2 min. 50 °C; 10 min. 95 °C; (15 s. 95 °C, 1 min. 58 °C) × 65. No specific amplification was detected, while positive controls with cDNA prepared with random hexamer primers and with genomic human DNA yielded HML-10(DAP3)-specific amplicons.

### Trypan Blue exclusion and cell viability (MTS) assays

Freshly passaged HeLa cells were seeded in 48-well plates (2x10^4^ cells per well in complete growth medium) and cultivated overnight to ~70% confluence. Cells were transfected with 50 nM of the indicated ASOs using the DreamFect Gold transfection reagent (OZ Biosciences) according to the manufacturer’s recommendations. Medium was replaced with fresh prewarmed complete growth medium 6 h post-transfection. At 24 h post-transfection, cells were stimulated with 1000 U/mL recombinant human IFNγ (PeproTech) or 100 ng/mL recombinant human TNFα (Biomol, Hamburg, Germany) for 24 h by addition to the growth medium, or left unstimulated. For Trypan Blue exclusion assays, cells were harvested 48 h post-transfection, resuspended in 50 μL PBS, mixed 1:1 (v/v) with 50 μL 0.4% (v/v) Trypan Blue Stain (Invitrogen) and incubated for 1 min. Total cell number and number of stained cells of each sample were counted in a hemocytometer. About 100–200 total cells per sample were counted. To obtain the fraction of dead cells, the number of stained cells was divided by the total cell number. For cell viability (MTS) assays, one tenth of the growth medium volume of MTS reagent (CellTiter 96 AQueous One Solution Cell Proliferation Assay by Promega) was added to each well 48 h post-transfection. Cells were incubated for approximately 1 h before the absorbance at 495 nm of the supernatants was measured with a NanoDrop ND-1000 Spectrophotometer (Thermo Scientific, Waltham, MA, USA). Fresh growth medium with the addition of one tenth of MTS reagent was used as blank.

### Detection of apoptosis by DNA laddering

Freshly passaged HeLa cells were seeded into 6-well plates (10^6^ cells per well in complete growth medium) and cultivated overnight to ~70% confluence. Cells were transfected with 50 nM of the indicated ASOs using the DreamFect Gold transfection reagent (OZ Biosciences) according to the manufacturer’s recommendations. Medium was replaced with fresh prewarmed complete growth medium 6 h post-transfection. At 48 h post-transfection, cells were lysed and DNA was prepared with the Apoptotic DNA Ladder Kit (Roche, Mannheim, Germany) according to the manufacturer’s recommendations. Samples were analyzed using a 1% agarose TAE gel and DNA was visualized with ethidium bromide.

## Additional files

Additional file 1: Figure S1. (**a**) Neighbor-joining trees of the flanking 1000 bp upstream (left tree) and downstream (right tree) relative to retroviral orientation. Two pairs of flanking sequences that cluster are highlighted by colored boxes, whereby elements nos. 3 and 43 only clustered in their upstream regions. Element numbers according to Table 1 are shown in parentheses. The horizontal bars represent 0.1 substitutions per nucleotide position. (**b**) Comparison of the genomic loci representing HML-10 elements nos. 3 and 43 (see blue boxes in panel (**a**)) that integrated into a *L1MB7* repeat or into a *MER4A1* repeat, respectively. Both HML-10 elements therefore likely represent independent integration events. (**c**) Comparison of the genomic loci representing HML-10 elements nos. 27 and 45 (red boxes in panel (**a**)) that are both flanked by *LTR5_Hs* and *HERVK-int* repeats in the same configuration. The overall sequence identity of the depicted regions is >88%. Therefore, one of these HML-10 was likely the result of a chromosomal duplication event that copied the other one. Images in panels (**b**) and (**c**) were obtained from the UCSC Genome Browser [68]. (PDF 1217 kb)
Additional file 2: Table S1. Presence or absence of HML-10 in different mammalian genomes. The number of *LTR14* hits that represent HML-10 elements in the indicated genomes were assessed with the UCSC Table Browser [67] by querying *LTR14* in the respective RepeatMasker tracks [69, 70]. BLAT [74] searches within the UCSC Genome Browser [68] using the consensus sequence of *LTR14* obtained from the DFAM database (www.dfam.org) [75] were performed verify the presence of HML-10. (PDF 262 kb)
Additional file 3: Figure S2. Identification of a putative TSS in the HML-10(DAP3) 5′LTR. The sequence shown is chr1:153935293–153936036 of the human genome hg18 assembly [1]. The HML-10(DAP3) 5′LTR (according to RepeatMasker annotation [70]) is highlighted in bold letters. Six Inr sequences highlighted blue were identified by sequence homology searches of the consensus YYANWYY sequence [76]. Five TATA boxes highlighted red were identified with the TFBind program [77] on http://tfbind.hgc.jp using a similarity threshold of 0.8. Inr2 and TATA4 are overlapping. Only one Inr element (Inr1) is located in close proximity downstream of a TATA box (TATA3). The putative TSS within Inr1 is underlined. A downstream promoter element (DPE) highlighted violet matching the consensus RGWYVT sequence [49] is located 19 bp downstream of the putative TSS. Primer locations for *LTRfor1*, *LTRfor2* and *LTRrev* (see Additional file 4) are indicated by arrows. An IFNγ activated sequence (GAS) is highlighted green. (PDF 364 kb)
Additional file 4: Protein fasta sequences of Pol proteins of different endogenous and exogenous betaretroviruses. (TXT 11 kb)
Additional file 5: Primer sequences. (XLS 42 kb)
Additional file 6: Sequences of ASOs. Asterisks denote phosphorothioate bonds. (XLS 39 kb)

## Abbreviations

5′RACE-PCR: 5′ rapid amplification of cDNA ends-PCR
ASO: Antisense oligonucleotide
BLAT: BLAST-like Alignment Tool
DAP3: Death-associated protein 3
DMEM: Dulbecco’s Modified Eagle’s Medium
DPE: Downstream promoter element
DR: Death Receptor
fLuc: Firefly luciferase
GAS: IFNγ activated sequence
HERV: Human endogenous retrovirus
HML: Human MMTV-like
HSV-TK: Herpes simplex virus thymidine kinase
HUVEC: Human umbilical vein endothelial cells
IFNγ: Interferon-gamma
Inr: Initiator element
JSRV: Jaagsiekte sheep retrovirus
LTR: long terminal repeat
MMTV: Mouse mammary tumor virus
MPMV: Mason-Pfizer monkey virus
Mya: Million years ago
NWM: New World monkey
OWM: Old World monkey
qRT-PCR: Quantitative real-time PCR
RE: Retroelement
rLuc: Renilla luciferase
RT: Reverse transcriptase
TE: Transposable element
TNFα: Tumor necrosis factor-alpha
TSD: Target site duplication
TSS: Transcription start site
