# Supplementary figures and images for: The intron-enriched HERV-K(HML-10) family suppresses apoptosis, an indicator of malignant transformation
Source: Mob DNA. 2016 Dec 7;7:25. doi: 10.1186/s13100-016-0081-9 (PMC5142424; doi:10.1186/s13100-016-0081-9)

**a**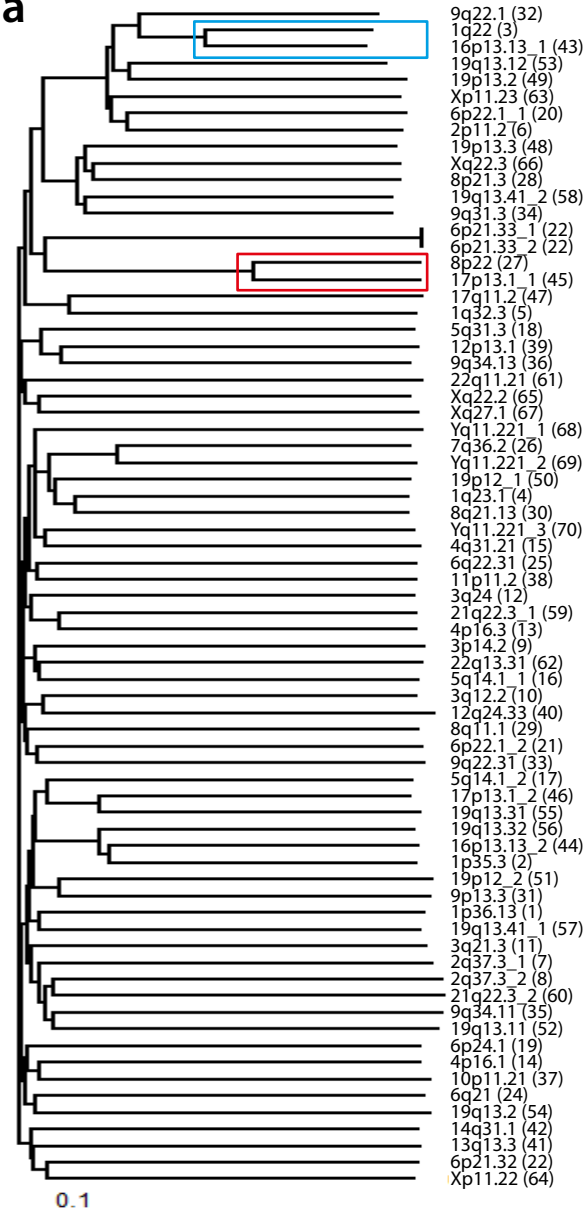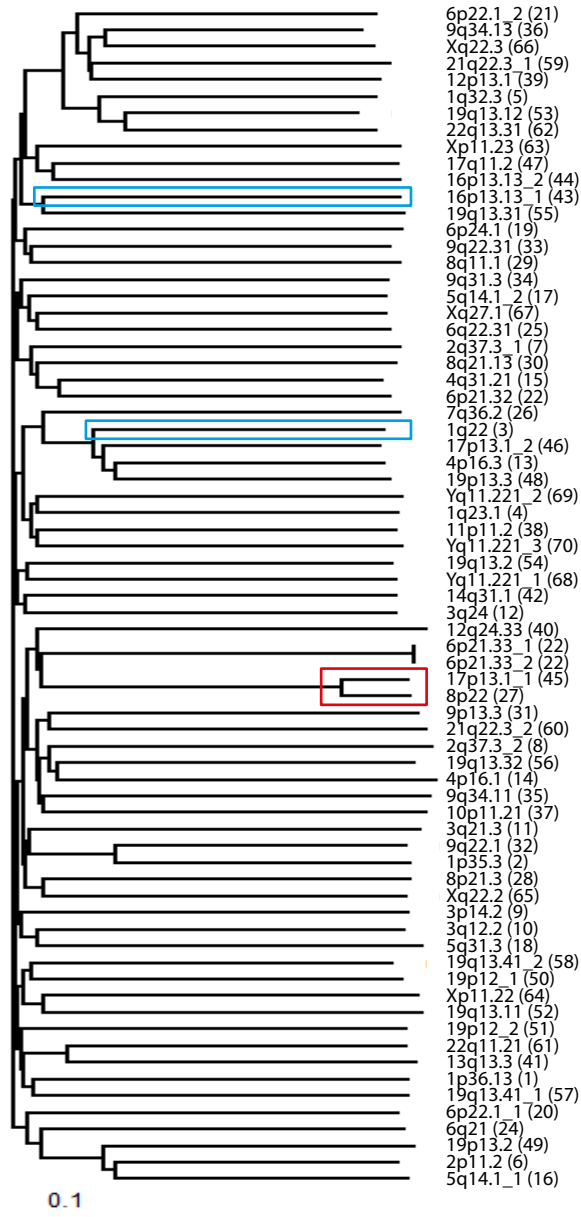**b**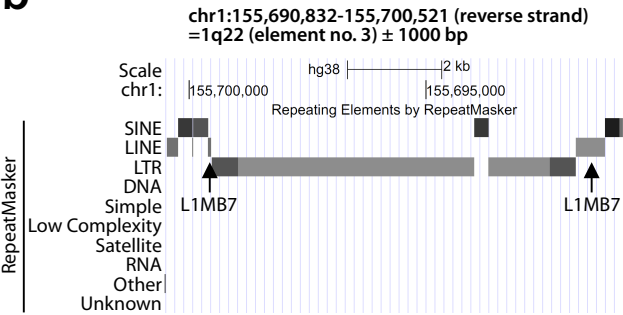**c**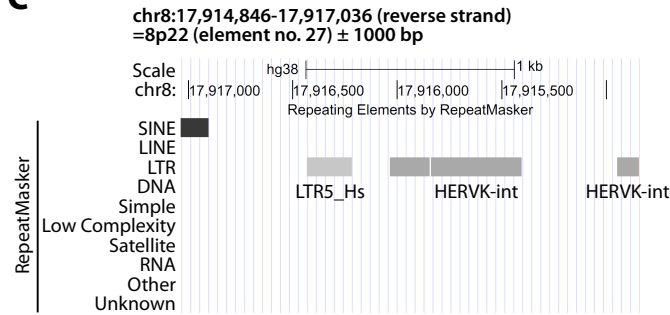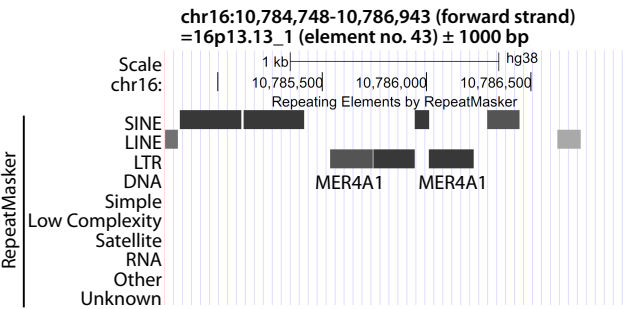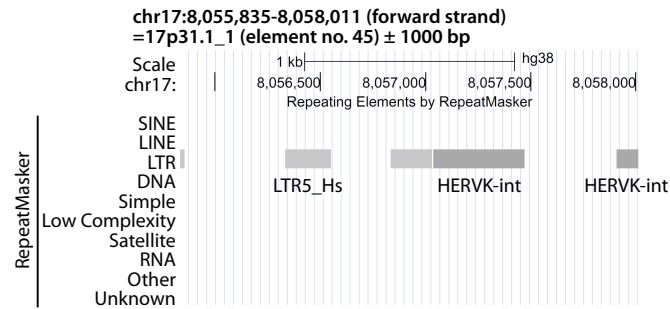

Supplement: Additional file 1: Figure S1. — (a) Neighbor-joining trees of the flanking 1000 bp upstream (left tree) and downstream (right tree) relative to retroviral orientation. Two pairs of flanking sequences that cluster are highlighted by colored boxes, whereby elements nos. 3 and 43 only clustered in their upstream regions. Element numbers according to Table 1 are shown in parentheses. The horizontal bars represent 0.1 substitutions per nucleotide position. (b) Comparison of the genomic loci representing HML-10 elements nos. 3 and 43 (see blue boxes in panel (a)) that integrated into a L1MB7 repeat or into a MER4A1 repeat, respectively. Both HML-10 elements therefore likely represent independent integration events. (c) Comparison of the genomic loci representing HML-10 elements nos. 27 and 45 (red boxes in panel (a)) that are both flanked by LTR5_Hs and HERVK-int repeats in the same configuration. The overall sequence identity of the depicted regions is >88%. Therefore, one of these HML-10 was likely the result of a chromosomal duplication event that copied the other one. Images in panels (b) and (c) were obtained from the UCSC Genome Browser [68]. (PDF 1217 kb) [file 13100_2016_81_MOESM1_ESM.pdf]
