# Supplementary material for: The intron-enriched HERV-K(HML-10) family suppresses apoptosis, an indicator of malignant transformation
Source: Mob DNA. 2016 Dec 7;7:25. doi: 10.1186/s13100-016-0081-9 (PMC5142424; doi:10.1186/s13100-016-0081-9)
Supplement: Additional file 2: Table S1. — Presence or absence of HML-10 in different mammalian genomes. The number of LTR14 hits that represent HML-10 elements in the indicated genomes were assessed with the UCSC Table Browser [67] by querying LTR14 in the respective RepeatMasker tracks [69, 70]. BLAT [74] searches within the UCSC Genome Browser [68] using the consensus sequence of LTR14 obtained from the DFAM database (www.dfam.org) [75] were performed verify the presence of HML-10. (PDF 262 kb) [file 13100_2016_81_MOESM2_ESM.pdf]

Genome	Assembly	*LTR14* hits	Max. BLAT score (% identity) (query: consensus *LTR14*)
Human	Dec. 2013 (GRCh38/hg38)	86	504 (96.2)
Chimp	Feb. 2011 (CSAC 2.1.4/panTro4)	89	504 (96.2)
Gorilla	May 2011 (gorGor3.1/gorGor3)	80	502 (96.0)
Orangutan	July 2007 (WUGSC 2.0.2/ponAbe2)	96	499 (96.7)
Gibbon	Jun. 2011 (GGSC Nleu1.1/nomLeu2)	83	498 (95.6)
Rhesus macaque	Oct. 2010 (BGI CR_1.0/rheMac3)	94	500 (95.8)
Baboon	Mar. 2012 (Baylor Panu_2.0/papAnu2)	88	475 (93.6)
Squirrel monkey	Oct. 2011 (Broad/saiBol1)	623	125 (80.9)
Marmoset	March 2009 (WUGSC 3.2/calJac3)	689	155 (86.8)
Tarsier	Aug. 2008 (Broad/tarSyr1)	0	24 (96.2)
Mouse lemur	Jul. 2007 (Broad/micMur1)	0	31 (97.0)
Bushbaby	Mar. 2011 (Broad/otoGar3)	0	29 (71.0)
Tree shrew	Dec. 2006 (Briad/tupBel1)	0	28 (63.4)
Mouse	Dec. 2011 (GRCm38/mm10)	0	23 (96.2)
