# Supplementary material for: The intron-enriched HERV-K(HML-10) family suppresses apoptosis, an indicator of malignant transformation
Source: Mob DNA. 2016 Dec 7;7:25. doi: 10.1186/s13100-016-0081-9 (PMC5142424; doi:10.1186/s13100-016-0081-9)
Supplement: Additional file 3: Figure S2. — Identification of a putative TSS in the HML-10(DAP3) 5′LTR. The sequence shown is chr1:153935293–153936036 of the human genome hg18 assembly [1]. The HML-10(DAP3) 5′LTR (according to RepeatMasker annotation [70]) is highlighted in bold letters. Six Inr sequences highlighted blue were identified by sequence homology searches of the consensus YYANWYY sequence [76]. Five TATA boxes highlighted red were identified with the TFBind program [77] on http://tfbind.hgc.jp using a similarity threshold of 0.8. Inr2 and TATA4 are overlapping. Only one Inr element (Inr1) is located in close proximity downstream of a TATA box (TATA3). The putative TSS within Inr1 is underlined. A downstream promoter element (DPE) highlighted violet matching the consensus RGWYVT sequence [49] is located 19 bp downstream of the putative TSS. Primer locations for LTRfor1, LTRfor2 and LTRrev (see Additional file 4) are indicated by arrows. An IFNγ activated sequence (GAS) is highlighted green. (PDF 364 kb) [file 13100_2016_81_MOESM3_ESM.pdf]

```
    AATGAAAAGGAAAAATATTTGAGTAGGCATTTCTCCAGAGTAGTTATACA

    AATGGCCCATAAGCACCTGAAAAGATGTTTAACATTACTAGTCATTAAGC

                                                TATA1
001 GTTGGGAAAAAGGCTCATGGGGTGCCTGCATAAACTGGCCATAAAAATAT

                  GAS
051 GGGACAATAAGTTGTGGAAAGCCACAAGAGGACTCTGAGGAGGAAAGTCT

101 TCTGATCACCATTATGTTCCCATGCTCTGAGCACAACCTGCTCTCTTATC

         TATA2                      LTRfor1
151 TATAAACACTGTGCTCAAGAAGAAAGACGCTCCTCTGAAGCATTGGAATG

              TATA3                     Inr1
201 TGGCTAGATATGCAGGCTCCTAGTTAAGCCCACTCCCACCAGCTAACTAA

                                          TATA4
251 AGATATGCTGCTTGAGCACAAAGGAGATTCACTTAAACCGCCTCTGCTAC
      DPE                                    Inr2

301 ACATTACGTGTATGACGTACTGCCTCCCTTTCACAGTTTTGCCCTGAACA

              Inr3          Inr4          TATA5
351 TCTGCTTCTTAGATCTAAGTGATTATACTCAATAAATAGTGTAGAGACCA

       LTRfor2                   Inr5
401 GAACTCTGGGCCTTTTGCAGCCTCCATTTTGCAACTGGCCCCTGGCTCC

                                         Inr6
451 CACCTTTATGAACTCTTAACCTGTCTCTTCTCATTCCTTTGTCGCCACCG

                             LTRrev
501 GACTTCAGGTACCCTACGGATGGTGTTGAGGCTGGTCCCCTACATTCTGG

    TGCCCAACATGAGGCCCGAAAGAATCCAGTGAAGGCATGCTCAAGCGTGT

    GAAATGGAGGACCGACAGACAAAGGACTCCCAAAGACGAAAAG
```
